# HLA allele and haplotype frequencies of registered stem cell donors in Chile

**DOI:** 10.3389/fimmu.2023.1175135

**Published:** 2023-05-29

**Authors:** Ute V. Solloch, Anette S. Giani, Maria Ignacia Pattillo Garnham, Jürgen Sauter, Stefanie N. Bernas, Vinzenz Lange, Francisco Barriga, Marcelo A. Fernández-Viña, Alexander H. Schmidt

**Affiliations:** ^1^ DKMS, Tübingen, Germany; ^2^ Fundación de Beneficencia Pública DKMS, Santiago, Chile; ^3^ DKMS Life Science Lab gGmbH, Dresden, Germany; ^4^ Department of Pathology, Stanford University, Stanford, CA, United States

**Keywords:** HSCT, HLA, haplotype frequency, donor registry, Chile

## Abstract

Patients in need of hematopoietic stem cell transplantation often rely on unrelated stem cell donors matched in certain human leukocyte antigen (HLA) genes. Donor search is complicated by the extensive allelic variability of the HLA system. Therefore, large registries of potential donors are maintained in many countries worldwide. Population-specific HLA characteristics determine the registry benefits for patients and also the need for further regional donor recruitment. In this work, we analyzed HLA allele and haplotype frequencies of donors of DKMS Chile, the first Chilean donor registry, with self-assessed “non-Indigenous” (*n*=92,788) and “Mapuche” (*n*=1,993) ancestry. We identified HLA alleles that were distinctly more abundant in the Chilean subpopulations than in worldwide reference populations, four of them particularly characteristic for the Mapuche subpopulation, namely B*39:09g, B*35:09, DRB1*04:07g, and DRB1*16:02g. Both population subsamples carried haplotypes of both Native American and European origin at high frequencies, reflecting Chile’s complex history of admixture and immigration. Matching probability analysis revealed limited benefits for Chilean patients (both non-Indigenous and Mapuche) from donor registries of non-Chilean donors, thus indicating a need for ongoing significant donor recruitment efforts in Chile.

## 1 Introduction

For many patients suffering from severe blood disorders, allogeneic hematopoietic stem cell transplantation (HSCT) is the best chance for a cure. Generally, siblings whose human leukocyte antigen (HLA) genes match those of the patient are the preferred stem cell donors, but only available for about 30% of patients (1). Other patients depend on a successful search for an unrelated donor in the global pool of registered potential stem cell donors.

In unrelated HSCT, the alleles of particular HLA genes have to be matched between patient and stem cell donor to avoid undesired reactions of donor immune cells against the host organism (2–4). *HLA* genes are located in the major histocompatibility complex (MHC) on the short arm of chromosome 6 and most of these genes are highly polymorphic. As of December 2022, 36,016 HLA alleles were described in the IPD-IMGT/HLA database (5). Because of the proximity of the gene loci in the MHC, the inheritance of HLA alleles is not stochastic but occurs in form of haplotypes with closely linked alleles. HLA allele and haplotype frequencies are population-specific, shaped for example by founder effects of early migration flows, mixing of gene pools of later migration movements, and reproductive and pathogen-driven modes of selection (6–9).

By the end of December 2022, the World Marrow Donor Association (WMDA) listed more than 40 million potential stem cell donors and cryopreserved cord blood units in their database (10). DKMS is a major donor registry with more than 11 million donors in seven countries. By the end of 2022, DKMS donors have donated hematopoietic stem cells from peripheral blood or bone marrow over 105,000 times to patients from 57 countries. In 2018, Fundación de Beneficencia Pública DKMS was founded in Chile. At the end of December 2022, it listed more than 165,000 potential donors.

In the Latin American countries, HSCT activity has been continuously increasing in recent years, but transplant rates and proportion of allotransplants from HLA-matched unrelated donors are still substantially lower compared with that in North America and European regions. The lower HSCT activity is related to the economic resources of the respective countries, but also to the insufficient representation of regional HLA genotypes in the global donor pool (11–14). In 2018, the founding year of Fundación de Beneficencia Pública DKMS, 212 hematopoietic cell transplants performed in Chile were reported to the Latin American Blood and Marrow Transplantation Group (LABMT) activity survey, 50.9% thereof in an allogenic setting. In only 18.5% (*n*=20) of these allogenic transplantations the donor was unrelated (14).

Chile is a country with a projected population of more than 19.5 million inhabitants in 2022 (15) in western South America, located between the Pacific Ocean in the west and Argentina in the east with the Andean Mountain Range as a natural border. The vast majority of the population inhabits the central third of the country around the capital of Santiago. As a result of occupation and immigration in the course of its history, the population of the present Chile has undergone several demographic shifts. Before the arrival of the Spanish conquerors in the middle of the 16^th^ century, Chile was inhabited by about twenty indigenous peoples, including the Mapuche, Aymara, Quechua and Easter Islanders (16). During the Spanish colonization, migration to Chile was initiated and controlled by the Spanish Empire. Mainly Spanish males migrated and mated with females from indigenous population groups (16, 17). Various indigenous tribes joined forces and succeeded to resist the conquerors in a long-running conflict and to defend parts of their territory in today’s Southern Chile until its occupation by the end of the 19th century (18). During the 19^th^ and 20^th^ century, immigration of different European populations took place after the declaration of independence in 1818; the Chilean government initiated selective entry of Europeans through colonization agencies. A large number of settlers immigrated from England, Ireland and Germany to Chile, but also from France, Italy, Greece, Croatia, and, more recently, from the Middle East (16, 19). Today, Chile is one of the main destination countries for migrants from South American and Caribbean countries (20).

In a 2020 survey, 52% of the Chilean population defined their ethnicity as “White”, 26% as “Mestizo”, 6% as “Indigenous”, and 2% as “Other”, while 14% did not classify themselves in any of these categories (21). A study on the genetic composition of Chilean individuals indicated predominantly European and Native American contributions with a minor African component (17). Ancestry proportions of individual subjects were found to be rather fluid than to occur in distinct steps that would differentiate different subpopulations. Several other studies have been conducted to describe the genetic structure of the Chilean population and its relationship to different South American Native and European populations based on HLA genes (9, 22–26).

In this study, we characterize HLA allele and haplotype frequencies of donors from Fundación de Beneficencia Pública DKMS (from here on referred to as “DKMS Chile”), the first Chilean stem cell donor registry. We highlight differences and similarities between the two major subpopulations of donors with self-assessed “Mapuche” or “non-Indigenous” origin. We further analyze the extent to which the Chilean population will benefit from the existence and further growth of the registry.

## 2 Subjects and methods

### 2.1 Donor samples and HLA typing

The DKMS Chile donor file included 126,512 potential stem cell donors with high-resolution genotyping for the HLA loci A, B, C, DRB1, DQB1 and DPB1 on March 29, 2022. 70.8% of the donors were female, and 47.2% were between 18 and 30 years old. Information on the ethnic origin of the donors was obtained by self-assessment during the registration process. For this purpose, donors were asked about the country of origin and ethnicity of both their parents. *n*=118,258 (93.5%) of the registered donors indicated Chile as both parents’ country of origin while the remaining 8,254 donors (6.5%) had at least one non-Chilean parent or did not provide corresponding information (**Supplemental Table 1**). Of the donors of Chilean origin, *n*=92,788 (78.5%) specified non-Indigenous as both parents’ ethnic origin (sample *CL-NI*). Also, 8,766 donors (7.4%) reported indigenous ancestry (**Supplemental Table 2**), including 1,993 (1.7%) with both parents belonging to the Mapuche ethnicity (sample *CL-MP*). Almost half of the donors (*CL-NI*: 47.7%; *CL-MP*: 49.9%) lived in the Santiago Metropolitan region. We analyzed the sets *CL-NI* and *CL-MP* in detail regarding allele frequency (AF) and haplotype frequency (HF).

For comparative analyses, we used 18 reference population samples (Argentinian, Bolivian, Brazilian, Colombian, English, French, German, US Hispanic, Indian, Irish, Italian, Mexican, Peruvian, Polish, Portuguese, Spanish, Turkish, and Venezuelan) with sample sizes between *n*=326 and *n*=100,000 individuals (**Supplemental Table 3**; this table includes also detailed information on the origin of the various population samples). Median and mean sample sizes were *n*=8,019 and *n*=33,707, respectively. In addition to South and Central American populations, we particularly considered populations with a connection to the migratory history of Chile (e.g., Spanish) but also some populations that are expected to have rather low genetic relatedness to Chilean individuals (e.g., Indian).

HLA genotyping of all donor samples was performed in a standardized amplicon-based next-generation sequencing workflow on Illumina platforms at DKMS Life Science Lab in Dresden, Germany. Primers were designed to target exons 2 and 3 of HLA-A, -B, -C, -DRB1, -DQB1 and -DPB1 (27, 28). DNA samples were obtained as buccal swabs with the informed consent of the donors.

### 2.2 Estimation of allele and haplotype frequencies

Allele frequencies (AF) were obtained by counting. Six-locus (HLA-A, -B, -C, -DRB1, -DQB1 and -DPB1) and 5-locus (HLA-A, -B, -C, -DRB1, and -DQB1) haplotype frequencies (HF) were calculated by the Hapl-o-Mat software (29, 30). Hapl-o-Mat estimates HF from population data using an expectation-maximization (EM) algorithm (31). HF provided by Hapl-o-Mat were truncated after a frequency of *f*=1/(2*n*) with *n* being the sample size, thus including only haplotypes with an expected occurrence of at least 1 in the sample. Prior to HF estimation, Hapl-o-Mat converts real-life donor HLA typing data to a homogenous resolution. The final HF and AF resolution levels in the present study are g-groups which combine alleles with synonymous mutations in the relevant exons (HLA class I genes: exon 2 and 3; HLA class II genes: exon 2) under a common allele group name denoted by a trailing “g” (32).

In order to identify alleles with a high specificity for Chilean populations, we defined large frequency difference (LFD) alleles similar to the study by Single et al. (9). For that purpose, we grouped 17 reference populations into four clusters: South and Central America (including Argentina, Bolivia, Brazil, Colombia, Mexico, Peru, and Venezuela), Southwestern Europe (France, Italy, Spain, and Portugal), Northern and Central Europe (England, Germany, Ireland, and Poland), and Others (India and Turkey). We omitted the US Hispanics as reference population because it comprises individuals of Central and South American – including Chilean – origin which would prevent the identification of HLA characteristics specific for Chileans. To qualify as an LFD allele of *CL-NI* or *CL-MP*, the respective AF in *CL-NI* or *CL-MP* had to be at least three times higher than the mean frequency of that allele in each of the four clusters.

### 2.3 Linkage disequilibrium, Hardy-Weinberg equilibrium

Based on the estimated 6-locus HF, the linkage disequilibrium (LD) coefficient *D’* was calculated for each two-locus allele combination of the donor samples *CL-NI* and *CL-MP* (33, 34). Significance was tested with Fisher’s exact test. *P*-values were subjected to Holm-Bonferroni correction for multiple testing. AF for the LD calculations were derived from haplotype frequencies and therefore may differ slightly from the AF determined by counting.

As the EM algorithm requires the underlying population to be in Hardy-Weinberg equilibrium (HWE), we tested for significant deviations from HWE utilizing an extension of Fisher’s exact test based on Guo and Thompson (35). The procedure was applied to the genotypes on g-group resolution level with Arlequin v 3.5 (36). Because large sample sizes as in this study bear the risk that tests indicate significant results without actual relevance (37), deviations from HWE were also evaluated *via* the effect size statistics *W_n_* (38) and by comparison of the observed and expected homozygosity for all population samples. *W_n_* values were calculated locus-wise on g-group resolution level. *W_n_* values range from 0 to 1, with values near 1 reflecting strong disequilibrium. Values below *W_n_* =0.1 were regarded as indicator for sufficient accordance with HWE. No correction for multiple testing was applied for HWE evaluations since this would bias the results towards accordance with HWE and lead to a loss of sensitivity.

### 2.4 Genetic distances

Genetic distances (GD) between the 20 samples (*CL-NI*, *CL-MP* plus 18 reference populations) were calculated as Cavalli-Sforza and Edwards chord distances (39) for the high-resolution AF of HLA loci A, B, C, DRB1, DQB1 and DPB1. Locus-wise chord distances *d_j_* were calculated using the formula $d_{j}=\frac{2}{\pi }\;\sqrt{2\cdot (1-\sum_{i=1}^{n}\sqrt{f_{i}\cdot g_{i}}})$ , where *n* is the total number of alleles and *f_i_* and *g_i_* are the AF of the two populations. The overall distance for each population pair was computed as $D=\sqrt{\sum_{j=1}^{m}d_{j}^{2}}$ , where *m* denotes the number of loci considered, i.e. *m*=6 in our case. Multidimensional scaling (MDS) was carried out in *R 3.6.3* (40) using the *cmdscale* function. The quality of the distance values’ fit to the graphical representation was evaluated with the goodness-of-fit (GOF) measure, which is based on the eigenvalues of the MDS solution and depends on the number of dimensions used. GOF can take a value between 0 and 1, with higher values indicating a better fit.

### 2.5 Matching probabilities

The probability for a patient from a given patient population to find at least one donor from a given donor population matching in HLA loci A, B, C, DRB1 and DQB1 was calculated as described before (32, 41, 42). We did not consider HLA-DPB1 in the matching probability analyses because a donor matched for the other 5 HLA genes included in this study (“10/10 match”) is usually accepted as a “full match”. To avoid an influence of different sample sizes on the calculated matching probabilities, we drew random samples of *n*=1,000 individuals from the populations. The analysis was conducted with 17 populations including *CL-NI*, *CL-MP* and all reference populations except Argentina, Bolivia, and Venezuela whose sample sizes were too small.

We calculated matching probabilities for three scenarios: (a) donor population *CL-NI*, 17 different patient populations (including *CL-NI* und *CL-MP*); (b) 17 donor populations, patient population *CL-NI*; and (c) 17 donor populations, patient population *CL-MP*. Due to the high proportion of non-Indigenous donors in DKMS Chile, the donor population *CL-NI* served as an approximation for the entire DKMS Chile donor file.

## 3 Results

### 3.1 AF and HF estimation

For the sample of non-Indigenous individuals (*CL-NI*), AF obtained by counting resulted in cumulative frequencies of the 10 most frequent alleles between 53.6% (HLA-B) and 96.1% (HLA-DQB1; **Table 1**, complete allele lists are given in **Supplemental Table 4**). The number of different alleles ranged from 35 (HLA-DQB1) to 205 (HLA-B). These results indicate a particularly high allelic diversity for HLA-B and a particularly low one for HLA-DQB1. Similar to the *CL-NI* sample, the cumulative frequency of the 10 most frequent alleles of the smaller *CL-MP* sample ranged from 57.5% (HLA-B) to 96.8% (HLA-DQB1; **Table 1**), while the number of alleles ranged from 19 (HLA-DQB1) to 100 (HLA-B; **Supplemental Table 5**). AF of the 18 reference populations are given in **Supplemental Table 6**.

**Table 1:** 10 most frequent HLA-A, -B, -C, DRB1, -DQB1 and -DPB1 alleles of non-Indigenous Chilean donors (sample *CL-NI*, *n*=92,788) and Mapuche donors (sample *CL-MP*, *n*=1,993).

CL-NI											
HLA-A		HLA-B		HLA-C		HLA-DRB1		HLA-DQB1		HLA-DPB1	
Allele	f	Allele	f	Allele	f	Allele	f	Allele	f	Allele	f
02:01g	0.2254	39:09g	0.0961	07:02g	0.1871	07:01g	0.1171	03:01g	0.2300	04:01g	0.2784
24:02g	0.1022	51:01g	0.0713	04:01g	0.1410	04:07g	0.0998	02:01g	0.1960	04:02g	0.2277
68:01g	0.1019	07:02g	0.0632	07:01g	0.1050	03:01g	0.0878	03:02g	0.1827	02:01g	0.1522
01:01g	0.0975	44:03g	0.0606	06:02g	0.0735	15:01g	0.0632	05:01g	0.0967	03:01g	0.0962
03:01g	0.0709	08:01g	0.0500	05:01g	0.0533	08:02g	0.0615	04:02g	0.0709	01:01g	0.0364
31:01g	0.0609	18:01g	0.0437	08:02g	0.0522	16:02g	0.0472	06:02g	0.0661	14:01g	0.0302
29:02g	0.0454	14:02g	0.0423	01:02g	0.0514	01:01g	0.0456	06:03g	0.0397	17:01g	0.0299
11:01g	0.0425	35:01g	0.0398	03:04g	0.0512	11:01g	0.0451	03:03g	0.0346	05:01g	0.0293
26:01g	0.0303	44:02g	0.0348	12:03g	0.0504	14:02g	0.0445	05:03g	0.0227	11:01g	0.0255
23:01g	0.0282	15:01g	0.0346	16:01g	0.0487	13:01g	0.0398	06:04g	0.0217	13:01g	0.0223
CL-MP											
HLA-A		HLA-B		HLA-C		HLA-DRB1		HLA-DQB1		HLA-DPB1	
Allele	f	Allele	f	Allele	f	Allele	f	Allele	f	Allele	f
02:01g	0.2298	39:09g	0.1626	07:02g	0.2479	04:07g	0.1448	03:01g	0.2662	04:02g	0.2790
68:01g	0.1505	51:01g	0.0652	04:01g	0.1468	07:01g	0.1051	03:02g	0.2173	04:01g	0.2474
24:02g	0.0998	07:02g	0.0562	07:01g	0.0931	08:02g	0.0808	02:01g	0.1698	02:01g	0.1375
01:01g	0.0775	44:03g	0.0479	06:02g	0.0675	16:02g	0.0738	04:02g	0.0878	03:01g	0.1257
31:01g	0.0660	35:09	0.0459	01:02g	0.0627	03:01g	0.0725	05:01g	0.0778	05:01g	0.0366
03:01g	0.0592	15:01g	0.0432	15:02g	0.0529	14:02g	0.0672	06:02g	0.0519	17:01g	0.0286
29:02g	0.0434	08:01g	0.0426	03:04g	0.0482	15:01g	0.0509	03:03g	0.0341	01:01g	0.0241
11:01g	0.0399	35:01g	0.0399	08:02g	0.0482	11:01g	0.0384	06:03g	0.0311	14:01g	0.0231
26:01g	0.0306	18:01g	0.0361	05:01g	0.0449	01:01g	0.0379	06:04g	0.0158	13:01g	0.0186
23:01g	0.0223	14:02g	0.0354	12:03g	0.0367	11:04g	0.0371	05:03g	0.0158	11:01g	0.0181

The 12 most frequent HLA alleles were identical for *CL-NI* and *CL-MP* in all 6 loci, with the following two exceptions: DRB1*09:01g (#15 in *CL-NI*), and DRB1*13:02g (#14 in *CL-MP*) (**Supplemental Table 4**). The 5 alleles with the largest absolute AF differences between the *CL-MP* and *CL-NI* samples were: B*39:09g (Δ*f*=0.066, *f_CL-NI_* =0.096, *f_CL-MP_* =0.163), C*07:02g (Δ*f*=0.061, *f_CL-NI_* =0.187, *f_CL-MP_* =0.248), DPB1*04:02g (Δ*f*=0.051, *f_CL-NI_* =0.228, *f_CL-MP_* =0.279), A*68:01g (Δ*f*=0.049, *f_CL-NI_* =0.102, *f_CL-MP_* =0.151), and DRB1*04:07g (Δ*f*=0.045, *f_CL-NI_* =0.100, *f_CL-MP_* =0.145). For each of these alleles, the frequency in the *CL-MP* sample was higher than in *CL-NI*. We observed the largest absolute AF difference between both samples with *f_CL-NI_* >*f_CL-MP_* (and eighth largest overall) for DPB1*04:01g (Δ*f*=-0.031, *f_CL-NI_* =0.278, *f_CL-MP_* =0.247).

When determining the largest ratios of AF of both population subsamples (the respective larger AF in the numerator), we considered only AF with a difference of at least 0.01 in absolute value in order to exclude random findings. With this constraint, the 5 alleles with the largest AF ratios were: B*39:09g (*f_CL-MP_ f_CL-NI_* =1.691, *f_CL-NI_* =0.096, *f_CL-MP_* =0.163), DRB1*16:02g (*f_CL-MP_ f_CL-NI_* =1.562, *f_CL-NI_* =0.047, *f_CL-MP_* =0.074), DRB1*14:02g (*f_CL-MP_ f_CL-NI_* =1.512, *f_CL-NI_* =0.044, *f_CL-MP_* =0.067), DPB1*01:01g (*f_CL-NI_ f_CL-MP_* =1.510, *f_CL-NI_* =0.036, *f_CL-MP_* =0.024), and B*35:09 (*f_CL-MP_ f_CL-NI_* =1.491, *f_CL-NI_* =0.031, *f_CL-MP_* =0.046). Similar to the analysis of the largest AF differences, the AF were higher in the *CL-MP* sample for 4 of 5 alleles, with the exception of DPB1*01:01g. The B*39:09g allele is not only the allele with the largest absolute AF difference and the largest AF ratio between both samples, but also the only one that is among the top 5 in both views.

Samples *CL-NI* and *CL-MP* comprised 6 (including 2 with *f*>0.01) and 8 (including 7 with *f*>0.01) LFD alleles, respectively (**Table 2**). Four of the aforementioned alleles with a considerable higher occurrence in *CL-MP* than in *CL-NI* (B*39:09g, B*35:09, DRB1*04:07g, and DRB1*16:02g) also were LFD alleles of *CL-MP* and had an AF>0.01 in this population. Thus, these are relevant alleles that are characteristic for the Mapuche population. Interestingly, we could not observe A*68:23 (43) in any of the reference populations apart from the US Hispanics though it was present in both *CL-NI* and *CL-MP*, in the latter even with an AF>0.01. The CIWD categories (Version 3.0.0 (44)) of the LFD alleles are given in **Supplemental Table 7**.

**Table 2:** Large frequency difference (LFD) alleles of the samples *CL-NI* and *CL-MP*.

	Sample	
Locus	*CL-NI*	*CL-MP*
A	68:16, *68:23*	68:16, *68:23*
B	**35:09**, 35:20, **39:09g**	**35:09**, **35:20**, **39:09g**
C	–	–
DRB1	**-**	**04:07g**, **16:02g**
DQB1	03:25	03:25
DPB1	–	–

The definition of LFD alleles is given in the Methods section. Bold: AF≥0.01. Underlined: AF remarkably higher than in CL-NI (among the 5 largest AF differences or ratios between CL-MP and CL-NI). Italic: Not observed in any of the non-Chilean reference populations (apart from the US Hispanics).

For the sample of non-Indigenous individuals, 6-locus HF estimation resulted in 19,451 haplotypes with a frequency of *f* ≥ 1/2*n*, summing up to a cumulated frequency of *f* = 0.980. The 20 most frequent HLA haplotypes of *CL-NI* had a cumulated frequency of *f* = 0.111 (**Table 3**; **Supplemental Table 8A**). HF estimation for the smaller Mapuche sample (*CL-MP*) resulted in 1,835 haplotypes with *f* ≥ 1/2*n* (cumulated frequency *f* = 0.984). The 20 most frequent haplotypes summed up to a cumulated frequency of *f* = 0.154 (**Table 3**; **Supplemental Table 9A**). The results of the 5-locus HF estimations are given in **Supplemental Table 8B** (*CL-NI*) and **Supplemental Table 9B** (*CL-MP).* Six-locus HF of the 18 reference populations are listed in **Supplemental Table 10**.

**Table 3:** 20 most frequent 6-locus HLA haplotypes of non-Indigenous Chilean donors (sample *CL-NI*, *n*=92,788) and Mapuche donors (sample *CL-MP*, *n*=1,993).

CL-NI							CL-MP						
HLA-A	HLA-B	HLA-C	HLA-DRB1	HLA-DQB1	HLA-DPB1	*f*	HLA-A	HLA-B	HLA-C	HLA-DRB1	HLA-DQB1	HLA-DPB1	*f*
02:01g	39:09g	07:02g	16:02g	03:01g	04:02g	0.01245	02:01g	39:09g	07:02g	16:02g	03:01g	04:02g	0.02213
68:01g	39:09g	07:02g	16:02g	03:01g	04:02g	0.00959	68:01g	39:09g	07:02g	16:02g	03:01g	04:02g	0.01963
01:01g	08:01g	07:01g	03:01g	02:01g	01:01g	0.00871	02:01g	39:09g	07:02g	04:07g	03:02g	03:01g	0.00912
29:02g	44:03g	16:01g	07:01g	02:01g	11:01g	0.00782	02:01g	07:02g	07:02g	15:01g	06:02g	04:01g	0.00820
01:01g	08:01g	07:01g	03:01g	02:01g	04:01g	0.00771	02:01g	39:09g	07:02g	08:02g	04:02g	04:02g	0.00817
03:01g	07:02g	07:02g	15:01g	06:02g	04:01g	0.00760	68:01g	39:09g	07:02g	14:02g	03:01g	04:02g	0.00733
68:01g	51:01g	15:02g	04:07g	03:02g	04:02g	0.00750	68:01g	51:01g	15:02g	04:07g	03:02g	04:02g	0.00714
02:01g	39:09g	07:02g	08:02g	04:02g	04:02g	0.00586	68:01g	39:09g	07:02g	08:02g	04:02g	04:02g	0.00674
24:02g	07:02g	07:02g	15:01g	06:02g	04:01g	0.00472	02:01g	39:09g	07:02g	14:02g	03:01g	04:02g	0.00665
02:01g	39:09g	07:02g	04:07g	03:02g	03:01g	0.00463	03:01g	07:02g	07:02g	15:01g	06:02g	04:01g	0.00663
68:01g	39:09g	07:02g	08:02g	04:02g	04:02g	0.00449	01:01g	08:01g	07:01g	03:01g	02:01g	04:01g	0.00637
30:02g	18:01g	05:01g	03:01g	02:01g	02:02g	0.00435	01:01g	38:01g	06:02g	11:04g	03:01g	02:01g	0.00575
02:01g	07:02g	07:02g	15:01g	06:02g	04:01g	0.00432	03:01g	49:01g	07:01g	11:02g	03:01g	03:01g	0.00552
29:02g	44:03g	16:01g	07:01g	02:01g	04:01g	0.00382	11:01g	08:01g	07:01g	03:01g	02:01g	04:01g	0.00547
01:01g	08:01g	07:01g	03:01g	02:01g	02:01g	0.00357	02:01g	39:09g	07:02g	08:02g	04:02g	03:01g	0.00526
33:01g	14:02g	08:02g	01:02g	05:01g	04:01g	0.00318	29:02g	44:03g	16:01g	07:01g	02:01g	04:01g	0.00516
68:01g	39:09g	07:02g	08:02g	04:02g	03:01g	0.00293	01:01g	08:01g	07:01g	03:01g	02:01g	01:01g	0.00496
68:02g	14:02g	08:02g	01:02g	05:01g	04:02g	0.00278	68:01g	15:01g	01:02g	04:07g	03:02g	04:02g	0.00475
02:01g	39:09g	07:02g	14:02g	03:01g	04:02g	0.00270	68:01g	39:09g	07:02g	08:02g	04:02g	03:01g	0.00467
01:01g	08:01g	07:01g	03:01g	02:01g	04:02g	0.00253	24:02g	39:09g	07:02g	14:02g	03:01g	04:02g	0.00463

Thirteen haplotypes were among the 20 most frequent for both samples, and the two most frequent haplotypes in each sample, namely A*02:01g~B*39:09g~C*07:02g~DRB1*16:02g~DQB1*03:01g~DPB1*04:02g and A*68:01g~B*39:09g~C*07:02g~DRB1*16:02g~DQB1*03:01g~DPB1*04:02g, were identical, albeit with considerably higher HF in *CL-MP* (see below). Seven of the 20 most frequent *CL-NI* haplotypes and 10 of the 20 most frequent *CL-MP* haplotypes included the partial haplotype B*39:09g~C*07:02g. The three haplotypes with the largest absolute HF differences between *CL-MP* and *CL-NI* were: A*68:01g~B*39:09g~C*07:02g~DRB1*16:02g~DQB1*03:01g~DPB1*04:02g (Δ*f*=0.0100, *f_CL-NI_* =0.0096, *f_CL-MP_* =0.0196), A*02:01g~B*39:09g~C*07:02g~DRB1*16:02g~DQB1*03:01g~DPB1*04:02g (Δ*f*=0.0097, *f_CL-NI_* =0.0124, *f_CL-MP_* =0.0221), and A*68:01g~B*39:09g~C*07:02g~DRB1*14:02g~DQB1*03:01g~DPB1*04:02g (Δ*f*=0.0051, *f_CL-NI_* =0.0022, *f_CL-MP_* =0.0073). These haplotypes include identical alleles for the HLA loci B, C, DQB1 and DPB1.

The three haplotypes with the largest HF ratios between the *CL-MP* and *CL-NI* samples (the respective larger HF in the numerator, only HF with a difference of at least 0.0025 in absolute value considered) were: A*11:01g~B*08:01g~C*07:01g~DRB1*03:01g~DQB1*02:01g~DPB1*04:01g (*f_CL-MP_* /*f_CL-NI_* =9.535, *f_CL-NI_* =0.0006, *f_CL-MP_* =0.0055), A*11:01g~B*07:02g~C*07:02g~DRB1*01:01g~DQB1*05:01g~DPB1*04:02g (*f_CL-MP_* /*f_CL-NI_* =4.533, *f_CL-NI_* =0.0007, *f_CL-MP_* =0.0033), A*01:01g~B*38:01g~C*06:02g~DRB1*11:04g~DQB1*03:01g~DPB1*02:01g (*f_CL-MP_* /*f_CL-NI_* =3.513, *f_CL-NI_* =0.0016, *f_CL-MP_* =0.0058)

(*f_CL-MP_ f_CL-NI_* =3.513, *f_CL-NI_* =0.0016, *f_CL-MP_* =0.0058). Interestingly, all three haplotypes are putatively of European origin. The first of these haplotypes has frequency rank #14 in *CL-MP*, but only rank #280 in *CL-NI*.

### 3.2 Linkage disequilibrium, Hardy-Weinberg equilibrium

LD was assessed for all allele pairs from the 6-locus haplotypes of donor samples *CL-NI* (*n*=137,185 pairs) and *CL-MP* (*n*=30,602 pairs). In *CL-NI*, there were 26 allele pairs with a 2-locus HF of *f* ≥ 0.01 and strong LD of *D’*≥ 0.9 among the 10,434 pairs in significant (*p*<0.05) LD (**Table 4**; see **Supplemental Table 11** for a list of allele pairs with significant LD and a haplotype representation of a least four counts). These 26 allele pairs included 16 DRB1~DQB1 and 10 B~C locus combinations. The pairs with the highest occurrence in the sample were DRB1*04:07g~DQB1*03:02g (*f*_obs_=0.0952) and B*39:09g~C*07:02g (*f*_obs_=0.0945). The highest *D’* values were observed for DRB1*10:01g~DQB1*05:01g (*D’*=0.9995), followed by DRB1*04:02g~DQB1*03:02g (*D’*=0.998) and DRB1*03:01g~DQB1*02:01g (*D’*=0.996). The allele pair B*39:09g~C*07:02g showed both a high frequency and a strong LD (*D’*=0.985).

**Table 4:** 2-locus linkage disequilibria of non-Indigenous Chilean donors (sample *CL-NI*, *n*=92,788).

Haplotype	*f*(ab) observed	*f*(a)	*f*(b)	D’	f(ab) expected	*p*	α(HB)
B*39:09g-C*07:02g	0.0945	0.0957	0.1848	0.9851	0.0177	0.00E+00	0.0022
B*07:02g-C*07:02g	0.0601	0.0623	0.1848	0.9554	0.0115	0.00E+00	0.0038
B*08:01g-C*07:01g	0.0464	0.0495	0.1032	0.9282	0.0051	0.00E+00	0.0071
B*14:02g-C*08:02g	0.0390	0.0418	0.0515	0.9290	0.0022	0.00E+00	0.0063
B*35:01g-C*04:01g	0.0374	0.0389	0.1379	0.9539	0.0054	0.00E+00	0.0042
B*35:09-C*04:01g	0.0300	0.0305	0.1379	0.9844	0.0042	0.00E+00	0.0023
B*40:01g-C*03:04g	0.0159	0.0169	0.0498	0.9354	0.0008	0.00E+00	0.0056
B*13:02g-C*06:02g	0.0140	0.0142	0.0721	0.9836	0.0010	1.41E-272	0.0100
B*52:01g-C*12:02g	0.0137	0.0170	0.0138	0.9948	0.0002	0.00E+00	0.0020
B*14:01g-C*08:02g	0.0114	0.0124	0.0515	0.9191	0.0006	2.05E-239	0.0125
DRB1*04:07g-DQB1*03:02g	0.0952	0.1791	0.0987	0.9564	0.0177	0.00E+00	0.0036
DRB1*03:01g-DQB1*02:01g	0.0864	0.1933	0.0867	0.9958	0.0168	0.00E+00	0.0019
DRB1*15:01g-DQB1*06:02g	0.0597	0.0651	0.0622	0.9578	0.0040	0.00E+00	0.0033
DRB1*16:02g-DQB1*03:01g	0.0454	0.2250	0.0467	0.9650	0.0105	0.00E+00	0.0028
DRB1*01:01g-DQB1*05:01g	0.0438	0.0948	0.0447	0.9777	0.0042	0.00E+00	0.0024
DRB1*11:01g-DQB1*03:01g	0.0428	0.2250	0.0439	0.9656	0.0099	0.00E+00	0.0026
DRB1*14:02g-DQB1*03:01g	0.0425	0.2250	0.0438	0.9631	0.0098	0.00E+00	0.0029
DRB1*13:01g-DQB1*06:03g	0.0368	0.0387	0.0388	0.9492	0.0015	0.00E+00	0.0050
DRB1*11:04g-DQB1*03:01g	0.0363	0.2250	0.0377	0.9516	0.0085	0.00E+00	0.0045
DRB1*01:02g-DQB1*05:01g	0.0290	0.0948	0.0300	0.9605	0.0028	0.00E+00	0.0031
DRB1*14:01g-DQB1*05:03g	0.0216	0.0223	0.0224	0.9697	0.0005	0.00E+00	0.0025
DRB1*13:02g-DQB1*06:04g	0.0209	0.0211	0.0307	0.9926	0.0006	0.00E+00	0.0021
DRB1*04:02g-DQB1*03:02g	0.0139	0.1791	0.0139	0.9981	0.0025	6.41E-180	0.0250
DRB1*10:01g-DQB1*05:01g	0.0121	0.0948	0.0121	0.9995	0.0011	3.09E-216	0.0167
DRB1*13:03g-DQB1*03:01g	0.0108	0.2250	0.0112	0.9532	0.0025	2.38E-114	0.0500
DRB1*15:02g-DQB1*06:01g	0.0104	0.0113	0.0105	0.9922	0.0001	1.37E-272	0.0083

Shown are all 26 significant allele pairs (p<0.05) with D’≥0.9 and f(ab)≥0.01. f(ab) = frequency of the partial haplotype; f(a) and f(b) = frequency of alleles a and b; D’=relative linkage disequilibrium; p = p-value reached by Fisher’s exact test; α(HB) = α-level after Holm-Bonferroni correction.

In the *CL-MP* sample, 872 pairs were in significant LD, including 24 with a 2-locus HF of *f* ≥ 0.01 and D’≥ 0.9 (**Table 5**; see **Supplemental Table 12** for a list of allele pairs with significant LD and a haplotype representation of a least four counts). Of these 24 pairs, 14 were DRB1~DQB1 and 10 B~C locus combinations. Again, the two most frequent allele pairs were B*39:09g~C*07:02g (*f*_obs_=0.1577) and DRB1*04:07g~DQB1*03:02g (*f*_obs_=0.1372), here with an even higher frequency than in *CL-NI*. B*52:01g-C*12:02g (*D’*=1) and B*40:01g-C*03:04g (*D’*=1) showed the strongest LD, followed by DRB1*01:01g-DQB1*05:01g (*D’*=0.993).

**Table 5:** 2-locus linkage disequilibria of Mapuche donors (sample *CL-MP*, *n*=1,933).

Haplotype	f(ab) observed	f(a)	f(b)	D’	f(ab) expected	*p*	α(HB)
B*39:09g-C*07:02g	0.1577	0.1613	0.2447	0.9710	0.0395	2.012E-38	0.0019
B*07:02g-C*07:02g	0.0544	0.0554	0.2447	0.9760	0.0136	1.859E-13	0.0033
B*35:09-C*04:01g	0.0438	0.0446	0.1432	0.9795	0.0064	7.164E-16	0.0029
B*08:01g-C*07:01g	0.0403	0.0423	0.0922	0.9477	0.0039	1.928E-17	0.0023
B*35:01g-C*04:01g	0.0358	0.0386	0.1432	0.9165	0.0055	1.845E-12	0.0036
B*14:02g-C*08:02g	0.0336	0.0351	0.0476	0.9550	0.0017	5.395E-17	0.0025
B*50:01g-C*06:02g	0.0147	0.0162	0.0664	0.9006	0.0011	1.982E-07	0.0071
B*13:02g-C*06:02g	0.0118	0.0123	0.0664	0.9562	0.0008	1.350E-06	0.0100
B*52:01g-C*12:02g	0.0115	0.0158	0.0115	1.0000	0.0002	1.118E-07	0.0063
B*40:01g-C*03:04g	0.0103	0.0103	0.0475	1.0000	0.0005	9.091E-07	0.0083
DRB1*04:07g-DQB1*03:02g	0.1372	0.2126	0.1420	0.9574	0.0302	1.109E-36	0.0020
DRB1*16:02g-DQB1*03:01g	0.0708	0.2632	0.0730	0.9580	0.0192	3.727E-16	0.0028
DRB1*03:01g-DQB1*02:01g	0.0706	0.1681	0.0719	0.9790	0.0121	2.957E-22	0.0022
DRB1*14:02g-DQB1*03:01g	0.0654	0.2632	0.0669	0.9695	0.0176	5.443E-15	0.0031
DRB1*15:01g-DQB1*06:02g	0.0483	0.0513	0.0503	0.9580	0.0026	1.090E-23	0.0021
DRB1*01:01g-DQB1*05:01g	0.0370	0.0764	0.0373	0.9927	0.0028	3.905E-17	0.0024
DRB1*11:01g-DQB1*03:01g	0.0358	0.2632	0.0378	0.9279	0.0099	8.294E-09	0.0050
DRB1*11:04g-DQB1*03:01g	0.0356	0.2632	0.0368	0.9537	0.0097	1.326E-08	0.0056
DRB1*13:01g-DQB1*06:03g	0.0289	0.0304	0.0307	0.9490	0.0009	1.360E-16	0.0026
DRB1*01:02g-DQB1*05:01g	0.0228	0.0764	0.0235	0.9654	0.0018	4.942E-11	0.0038
DRB1*04:04g-DQB1*03:02g	0.0194	0.2126	0.0204	0.9374	0.0043	3.327E-06	0.0125
DRB1*13:02g-DQB1*06:04g	0.0148	0.0158	0.0238	0.9350	0.0004	1.681E-09	0.0045
DRB1*14:01g-DQB1*05:03g	0.0146	0.0156	0.0153	0.9500	0.0002	1.681E-09	0.0042
DRB1*04:02g-DQB1*03:02g	0.0127	0.2126	0.0129	0.9753	0.0027	1.267E-04	0.0167

Shown are all 24 significant allele pairs (p<0.05) with D’≥0.9 and f(ab)≥0.01. f(ab) = frequency of the partial haplotype; f(a) and f(b) = frequency of alleles a and b; D’=relative linkage disequilibrium; p = p-value reached by Fisher’s exact test; α(HB) = α-level after Holm-Bonferroni correction.

The sample of non-Indigenous Chilean individuals (*CL-NI*) displayed significant deviations from HWE for four HLA loci (A, B, C and DQB1) in the exact test, the Mapuche sample (*CL-MP*) for three HLA loci, namely B, C and DRB1 (**Table 6**, see **Supplemental Table 13** for results of HWE tests for all 20 population samples). However, effect size values *W_n_* were generally small with *W_n_* =0.024 for HLA-B in *CL-MP* being the highest observed value, thus indicating only moderate deviations from HWE. Furthermore, we observed an excess homozygosity for all loci, and it has been shown that deviations from HWE towards excess homozygosity are not detrimental for HF estimation using the EM algorithm (45). Taken together, the results of the HWE tests are not a constraint for the analyses performed in this work with samples *CL-NI* and *CL-MP*.

**Table 6:** Results of Hardy-Weinberg equilibrium tests for non-Indigenous Chilean donors (sample *CL-NI*, *n*=92,788) and Mapuche donors (sample *CL-MP*, *n*=1,993).

Sample	Locus	*p*	*W_n_*	Observed homozygosity	Expected homozygosity
*CL-NI*	A	0.02014	0.00082	0.09994	0.09804
	B	0.00000	0.00106	0.04349	0.03946
	C	0.03619	0.00049	0.09420	0.09158
	DRB1	0.36453	0.00045	0.05956	0.05609
	DQB1	0.00388	0.00021	0.15125	0.14758
	DPB1	0.06840	0.00037	0.17361	0.16777
*CL-MP*	A	0.16790	0.01246	0.11189	0.10634
	B	0.04426	0.02444	0.06322	0.05277
	C	0.03798	0.00862	0.12895	0.11370
	DRB1	0.00108	0.01574	0.07376	0.06548
	DQB1	0.06787	0.00685	0.16809	0.16627
	DPB1	0.57292	0.00526	0.18214	0.17820

For each locus, measures of Fischer’s exact test (p), the effect size statistics W_n_ and observed and expected homozygosities are shown.

### 3.3 Genetic distances

**Figure 1:**
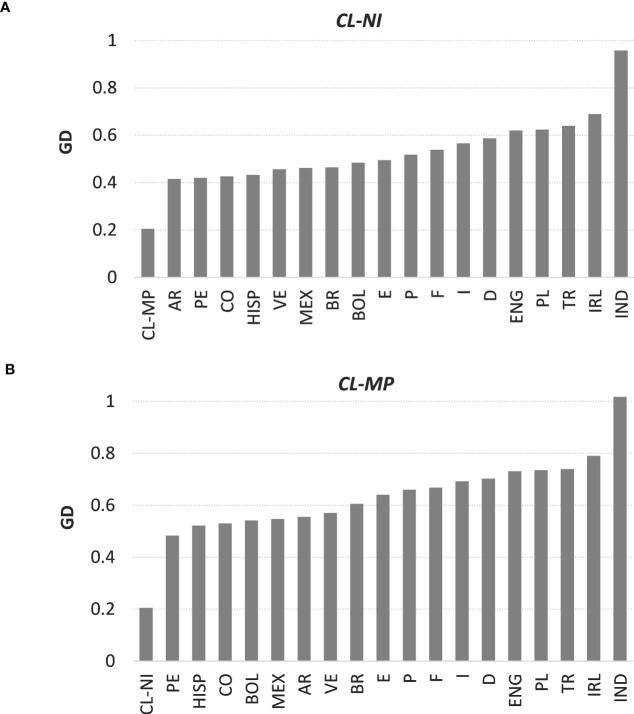
Genetic distances of samples **(A)** *CL-NI* and **(B)** *CL-MP* to the respective other 19 population samples. Shown are Euclidian overall values of Cavalli-Sforza and Edwards chord distance over the six loci HLA-A, -B, -C, DRB1, -DQB1, and -DPB1. Abbreviations for the populations: *CL-NI*=Chile non-Indigenous, *CL-MP*=Chile Mapuche, *AR*=Argentinian, *BOL*=Bolivian, *BR*=Brazilian, *CO*=Colombian, *D*=German, *E*=Spanish, *ENG*=English, *F*=French, *HISP*=US Hispanics, *I*=Italian, *IND*=Indian, *IRL*=Irish, *MEX*=Mexican, *P*=Portuguese, *PE*=Peruvian, *PL*=Polish, *TR*=Turkish, *VE*=Venezuelan.

The overall GD between non-Indigenous and Mapuche individuals from Chile was *d*=0.205 (see **Supplemental Table 14** for GD overall and single-locus results, **Figure 1** as graphical representation of overall distances of *CL-NI* and *CL-MP* to the respective other populations). This was the fourth smallest distance we observed, behind the distances between France and Germany (*d*=0.157), Portugal and Spain (*d*=0.168), and England and Ireland (*d*=0.175). Apart from the distance to the respective other Chilean subpopulation, *CL-NI* had the smallest GD to Argentina (*d*=0.416), Peru (*d*=0.420) and Colombia (*d*=0.427), *CL-MP* to Peru (*d*=0.483), the US Hispanics (*d*=0.522) and Colombia (*d*=0.530). We observed the largest distances of *CL-NI* and *CL-MP* to India (*d*=0.958 for *CL-NI*, *d*=1.017 for *CL-MP*), Ireland (*d*=0.690 for *CL-NI*, *d*=0.790 for *CL-MP*), and Turkey (*d*=0.639 for *CL-NI*, *d*=0.740 for *CL-MP*). For each of the 18 reference populations, the distance to *CL-NI* is smaller than to *CL-MP*. Distances calculated for the individual loci followed the described general pattern of the overall GD, whereby the order of the respective populations varied in detail.

**Figure 2:**
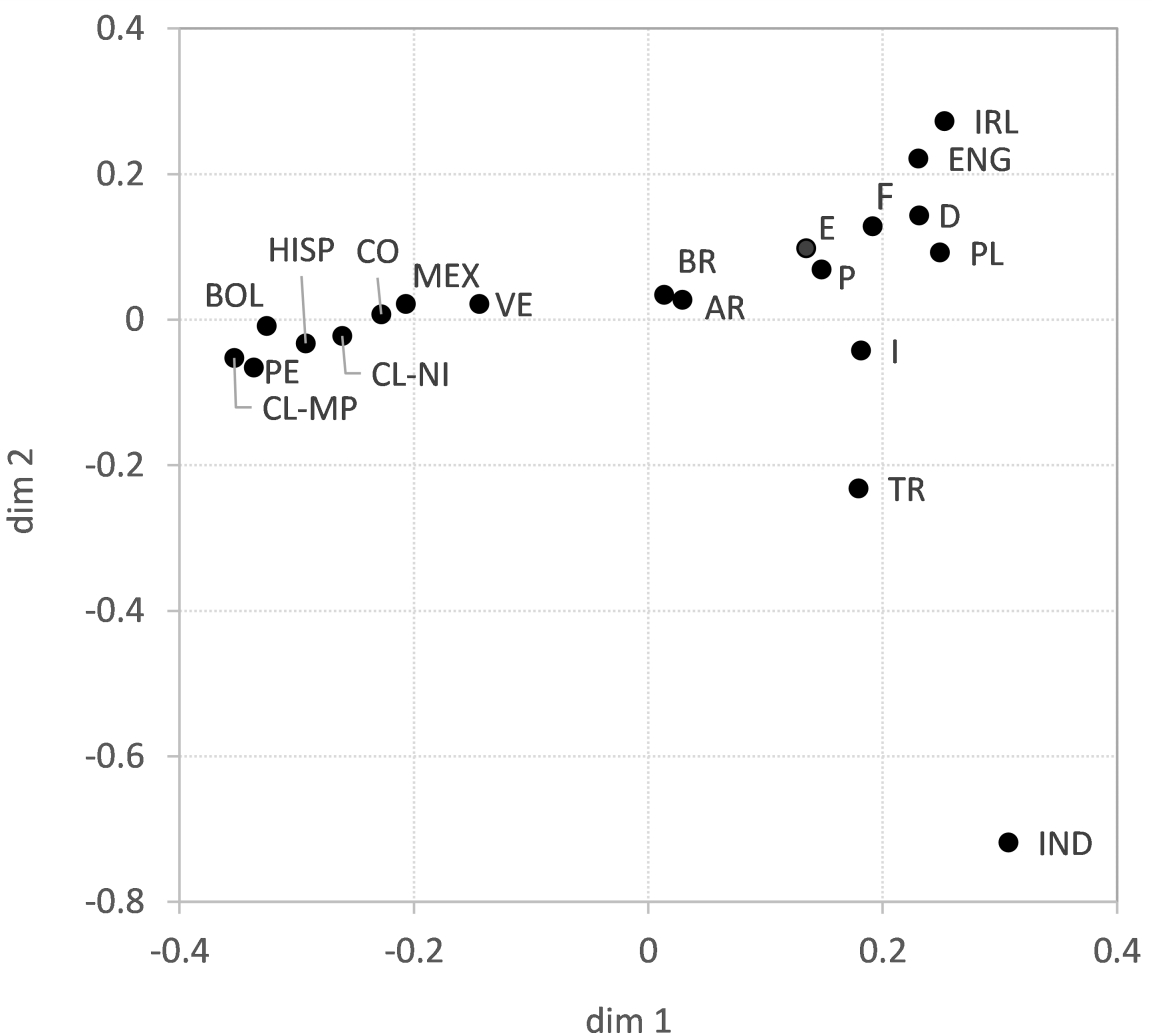
Genetic distances (Euclidian overall distances computed from single-locus Cavalli-Sforza and Edwards chord distances) of samples *CL-NI*, *CL-MP* and 18 reference population samples visualized by multidimensional scaling. Dimensions 1 and 2 explain 33.3% and 23.6% of the variance, respectively. Abbreviations for the populations: *CL-NI*=Chile non-Indigenous, *CL-MP*=Chile Mapuche, *AR*=Argentinian, *BOL*=Bolivian, *BR*=Brazilian, *CO*=Colombian, *D*=German, *E*=Spanish, *ENG*=English, *F*=French, *HISP*=US Hispanics, *I*=Italian, *IND*=Indian, *IRL*=Irish, *MEX*=Mexican, *P*=Portuguese, *PE*=Peruvian, *PL*=Polish, *TR*=Turkish, *VE*=Venezuelan.

The above results suggest a decisive influence of geographic location on GD. This is supported by the two-dimensional GD visualization using multidimensional scaling (**Figure 2**). In the upper right part of the figure, the European populations cluster, with the Southern European populations being closer to the Southern and Central American populations than those from Western and Central Europe. The larger distances from *CL-MP* to the reference populations compared with *CL-NI* are also evident here, with the exception of the populations from Bolivia and Peru, which are closer to *CL-MP* in the figure. Also, the relatively close proximity of *CL-NI* and *CL-MP* to Argentina, evident in the genetic distances, is not appropriately reflected in the two-dimensional MDS. The GOF value of the shown visualization is 0.569 (Dimensions 1 and 2 explain 33.3% and 23.6% of the variance, respectively) thus indicating only a moderate fit of the data.

### 3.4 Matching probabilities

**Figure 3:**
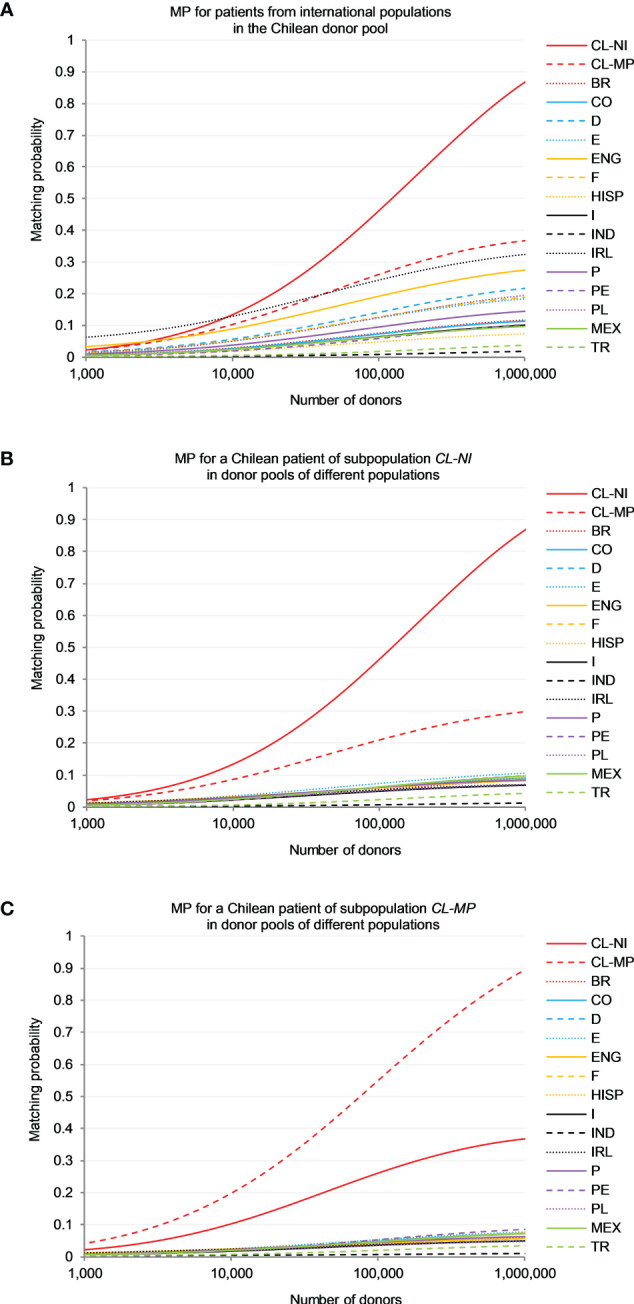
Matching probability (MP) to find a 10/10-matched unrelated donor. **(A)** MP for patients from different international populations in the Chilean donor pool (sample *CL-NI*). **(B)** MP for a Chilean patient of subpopulation *CL-NI* in donor pools of different populations. **(C)** MP for a Chilean patient of subpopulation *CL-MP* in donor pools of different populations. Abbreviations for the populations: *CL-NI*=Chile non-Indigenous, *CL-MP*=Chile Mapuche, *BR*=Brazilian, *CO*=Colombian, *D*=German, *E*=Spanish, *ENG*=English, *F*=French, *HISP*=US Hispanics, *I*=Italian, *IND*=Indian, *IRL*=Irish, *MEX*=Mexican, *P*=Portuguese, *PE*=Peruvian, *PL*=Polish, *TR*=Turkish.

5-locus (HLA-A, -B, -C, -DRB1 and -DQB1) high-resolution MP were calculated for patients from 15 reference populations searching in the Chilean donor pool, represented by population sample *CL-NI* (**Figure 3A**), and for non-Indigenous (*CL-NI*, **Figure 3B**) and Mapuche (*CL-MP*, **Figure 3C**) patients searching in virtual pools of donors from these 15 populations (see **Supplemental Table 15** for MP values at different registry sizes). In the following description, all MP values refer to a donor registry size of *n*=100,000 unless specified otherwise.

For patients from both Chilean subpopulations the chances of finding a matching stem cell donor were by far greatest in a registry of donors from their own population (e.g. *p*=0.465 for *CL-NI* and *p*=0.554 for *CL-MP*; **Figures 3B, C**), followed by a registry of donors from the respective other Chilean subpopulation (*p*=0.210 for *CL-NI* patients and *CL-MP* donors and *p*=0.263 for the opposite scenario).

In a direct comparison of the two matching scenarios with specific focus on *CL-NI* individuals (**Figures 3A, B**), it was evident that the MP curves of the reference populations for the scenario with *CL-NI* donors spread out significantly more than the corresponding curves for the scenario with *CL-NI* patients. For example, for a *CL-NI* donor registry the largest MP observed for non-Chilean reference populations were *p*=0.245 (Ireland), *p*=0.193 (England) and *p*=0.142 (Germany). In contrast, the three largest MP occurring for *CL-NI* patients searching in donor registries with non-Chilean donors were p=0.073 (Spain), p=0.064 (Mexico) and p=0.062 (Colombia, France, Portugal and Peru), i.e., significantly lower. It was also noticeable that the countries with particularly high MP values in the scenario with *CL-NI* donors, e.g., Ireland, England, and Germany, have rather large GD to *CL-NI* (**Figure 1**; **Supplemental Table 14**). This is counterintuitive to some extent because one might expect registered donors to be particularly beneficial for patients from genetically closely related populations.

To understand the mechanisms underlying these results, it is helpful to look at the genotypes that can be formed for each population from the population-specific haplotype frequencies. The cumulative frequency of genotypes shared between donor and patient population in the patient population is the upper limit for the MP, because for patients with a genotype not present in the donor population, in principle no matching donor can be found in this population. With increasing registry size, the MP approach this upper limit. Therefore, there is a high correlation between MP and cumulative frequencies of shared genotypes in the patient populations, especially for large donor registry sizes. In our data, this holds for the scenario “Donors from *CL-NI*, patients from various populations” (Spearman’s ρ=0.990; **Figure 3A**) as well as for the scenario “Donors from various populations, patients from *CL-NI*” (Spearman’s ρ=0.934; **Figure 3B**) at registry size *n*=100,000.

For further illustration, we use the example of the *CL-NI* and Irish (*IRL*) populations (**Supplemental Table 16**). Of all populations considered, the fewest genotypes can be formed for *IRL* from the haplotypes obtained with the EM algorithm, namely *g*=228,127. Since the sample size in this analysis was uniform for all populations, genotype number and genetic diversity are positively correlated. The corresponding value for *CL-NI* is *g*=474,985 while the number of shared genotypes is *g*=10,362. These shared genotypes have a cumulative frequency of *f*=0.338 in the *IRL* population, but only of *f*=0.073 in *CL-NI*. The low intra-population diversity of our *IRL* sample and the fact that the shared genotypes have a significantly higher frequency in *IRL* than in *CL-NI* are well in line with the insularity of Ireland and the significant migration from Ireland to Chile. Consistent with the cumulative frequencies of the shared genotypes, the *MP* for *CL-NI* donors and *IRL* patients is *p*=0.245 for *n*=100,000 and *p*=0.325 for *n*=1,000,000. In the opposite scenario with *IRL* donors and *CL-NI* patients, the corresponding values are *p*=0.054 (*n*=100,000) and *p*=0.071 (*n*=1,000,000).

## 4 Discussion

From a data set of 126,512 potential hematopoietic stem cell donors registered with DKMS Chile, we analyzed two population subsets, namely donors with self-assigned non-Indigenous (*CL-NI*, *n*=92,788) and Mapuche (*CL-MP, n*=1,993) origin. We examined specific characteristics of HLA allele and haplotype distributions based on typing information of the loci A, B, C, DRB1, DQB1 and DPB1 and related it to samples from 18 reference populations.

Previous knowledge of Chilean HLA AF and HF is limited and mostly restricted to data from rather small population samples of confined regions. The so far largest of the studies analyzed genotypic data of *n*=920 umbilical cord blood units of donors from Santiago with predominantly Western European ancestry (25). This analysis is limited to loci HLA-A and -B at low (antigenic) resolution and HLA-DRB1 at high resolution. When comparing the results at this resolution level, we see good agreement between the AF of the cord blood study and the *CL-NI* subpopulation in this work. For example, for each HLA locus the five most frequent alleles are identical. However, the allele frequency differences vary between 0.2% (B*35) and 39.3% (DRB1*04:07g). The closest concordance of AF is seen in locus HLA-A. The two most frequent haplotypes of both population samples are the same, while the third most frequent haplotype in the previous study (A*02~B*39~DRB1*08:02) only reaches rank 7 in this study, and our third most frequent haplotype (A*02~B*39~DRB1*16:02) ranks 12th in the cord blood bank study. A further study counted high-resolution HLA-DRB1 AF on a Chilean population of *n*=510 individuals from Talca, a city 250 km south of Santiago, and HLA-A, -B, and -C high resolution AF for *n*=160 thereof (26). The agreement with the AF of the *CL-NI* subpopulation in our study is only moderate. The Top 3 alleles are identical for loci HLA-A and -C, but with a change in rank order between alleles A*68:01g and A*24:02g (Δ*f*= 1.4% and -0.2%, respectively). Loci HLA-B and -DRB1 show greater differences in allele ranking and frequency. Two additional studies focusing specifically on Mapuche HLA allele and haplotype distributions in samples of *n*=104 and *n*=66 individuals from Cañete, a city in the Biobío region 600 km south of Santiago, found the same prevalent alleles as we did in our *CL-MP* sample, but some frequencies differ considerably (23, 24). While, for example, DRB1*04:07g is the most frequent DRB1 allele (*f*=13.9%) in our study, it only reaches 2.9% and 3.0% in the former studies. In contrast, the DRB1*04:03 allele, found at remarkably high frequencies of 31.7% (24) and 18.4% (23), had only an AF of *f*=1.2% in our study. In another study with a small sample of *n*=20 Huilliche, a Mapuche subgroup in southern Chile (9), DRB1*04:07g was again the most frequent DRB1 allele with *f*=27.5%. The comparison between our study and the former Mapuche population studies is hampered by the different sampling approaches. The previous studies were limited to very small and geographically restricted groups of individuals, whereas our study includes all donors from DKMS Chile who reported Mapuche origin for both parents and thus may represent an admixture of different Chilean Mapuche subpopulations. Previous studies discussed that discrepancies in allele frequencies may also result from nonrandom mating in isolated groups (46, 47).

Our *CL-MP* sample included 8 LFD alleles, namely A*68:16, A*68:23, B*35:09, B*35:20, B*39:09g, DRB1*04:07g, DRB1*16:02g and DQB1*03:25. All of these, with the exception of the two DRB1 alleles, were also LFD alleles of the *CL-NI* sample. We identified 4 of the LFD alleles (B*35:09, B*39:09g, DRB1*04:07g and DRB1*16:02g) as relevant and characteristic for our Mapuche sample because their AF in *CL-MP* were – with the minimum value of *f*=0.046 for B*35:09 – substantially greater than *f*=0.01 and particularly different from that in *CL-NI*. B*39:09g and DRB1*04:07g were even the most frequent alleles of their respective locus with *f*=0.163 and *f*=0.145, respectively.

Our definition of LFD alleles is similar to that of Single et al. (9). Nevertheless, due to the different choice of references, there are also clear differences between the two approaches. We intended to identify alleles that are particularly typical of Chile, i.e., that stand out in frequency from other populations, including South and Central American populations. In contrast, the earlier study aimed to identify alleles that are considerably more frequent in Native American populations than in other world regions. The fact that the LFD alleles identified in our study - the exception DQB1*03:25 is due to the fact that the Single et al. study did not include the DQB1 locus - are a subset of the LFD alleles identified in the earlier study suggests that the alleles are Native American in origin. A*68:16 and A*68:23, e.g., identified by us as LFD alleles of *CL-MP* and *CL-NI*, were described by Single et al. as “endemic”, i.e. absent from all world regions outside the Americas. This is confirmed by our results, as we found them outside Chile only in the Peruvian (A*68:16) and US Hispanics (both A*68:16 and A*68:23) population samples.

The choice of our reference populations was also driven by the number of registered DKMS donors in the different populations. In particular, the absence of other Native American reference populations makes it difficult to identify specific Mapuche alleles. Similarly, the absence of sub-Saharan West African populations prevents the assessment of an African contribution to today’s Chilean population, which, however, is estimated to be very minor (17). To balance this deficiency, we examined the global occurrences and frequencies of our LFD alleles in other populations in the literature and the Allele Frequency Net Database (AFND), a repository for immune-related gene polymorphisms in worldwide populations (48). Alleles included in our g-groups were considered.

Four of our 8 LFD alleles (A*68:16, A*68:23, B*35:09, DQB1*03:25) have been documented to date exclusively in South or Central American Native populations. In addition to the description of a respective unique occurrence in a Chilean Huiliche population (9), A*68:16 and A*68:23 were found in Chilean Mapuche (23) and in Chimila Amerindians of Colombia (49). Another study, whose results are not documented in AFND, reported the finding of allele A*68:23 in a cohort of *n*=559 Chileans from Santiago with a frequency of *f*>0.0125 (43). The highest AF of DQB1*03:25 in AFND has so far been reported in a population from Colombia Bogotá Cord Blood with *f*=0.0003, which corresponds to the presence of one allele in the sample of *n*=1,463 (50). B*35:09 has been reported with the highest frequencies in a Chilean Huiliche (f=0.075 (9)) and a Quechua population living in Bolivia and Peru (*f*=0.071 (9)). Further substantial occurrence was published for a Wichí population of Argentina (*f*=0.033 (51)).

The four remaining LFD alleles show - in addition to their occurrence in South and Central American indigenous populations - also relevant incidences in other world regions. B*35:20 has been documented before in a Mapuche population sample (*f*=0.024 (23)) and several other South and Central American Native populations, for example in two Colombian Ticuna populations (*f*=0.079 and *f*=0.036 (9)) and a Mestizo population from Nicaragua (f=0.013 (52)), but also occurs in other world regions, e.g. India (*f*=0.037 (53)), Israel (*f*=0.035 (54)), and Singapore (*f*=0.012 (54)). B*39:09g stands out in our analyses as the most frequent HLA-B allele and by a considerably higher frequency in *CL-MP* (*f*=0.163) than in the non-Indigenous *CL-NI* (*f*=0.096). To AFND, this allele was reported at very high frequencies in the above described Huiliche (*f*=0.425 (9)) and Mapuche population samples (*f*=0.200 (23)) from Chile and in a Yucpa population from Venezuela (*f*=0.349 (55)). Apart from further South American populations, occurrence of the allele, albeit with much lower incidence, was reported from several Asian, e.g. Thai (*f*=0.031 (56)) and Chinese (*f*=0.015 (57)) populations. DRB1*04:07g is not only abundant in South American populations with the highest occurrence in a Kogi population in Colombia (*f*=0.613 (58)), but also documented in virtually all American Native populations, for example, South Dakota Lakota Sioux (*f*=0.188 (59)) and Alaska Native or Aleut (*f*=0.020 (60)). The allele was further found in numerous European population samples. A similar abundance across American indigenous populations such as e.g. Colombian Sikuani (*f*=0.389 (58)), New Mexico Zuni (*f*=0.190 (54);) and Alaska Native or Aleut (*f*=0.371 (60)) is seen for DRB1*16:02g. Beyond that, the allele is frequent in Pacific and Southeast Asian populations, e.g. from Papua New Guinea (*f*=0.288 (61)), Malaysia (*f*=0.280 (62)), and Thailand (*f*=0.141 (63)).

In the HLA haplotypes, the LFD alleles frequently occur combined in conserved associations. 10 of the 20 most frequent 6-locus HLA haplotypes of the *CL-MP* sample contain B*39:09g (including the 3 most frequent and 5 of the 6 most frequent), always in combination with C*07:02g. The Native American alleles DRB1*04:07g and DRB1*16:02g are found in 3 and 2 of the 20 most frequent *CL-MP* haplotypes, respectively. DRB1*16:02g is predominantly associated with B*39:09g~C*07:02g and DQB1*03:01, while DRB1*04:07g~DQB1*03:02 also occurs with other B~C allele combinations. Despite their self-assessed non-Indigenous family background, donors of population sample *CL-NI* also carry a number of haplotypes containing blocks of frequent South and Central American indigenous alleles. For example, 7 of the 20 most frequent *CL-NI* haplotypes share the block B*39:09g~C*07:02g, including the two most frequent, which are identical to the two most frequent haplotypes in the *CL-MP* sample. Haplotypes with clear European origin are also found with high frequencies in both Chilean subsamples. For example, partial haplotypes B*08:01g~C*07:01g~DRB1*03:01g~DQB1*02:01g (frequency ranks 3, 5, 15 and 20 in *CL-NI* and 11, 14, and 17 in *CL-MP*) and B*07:02g~C*07:02g~DRB1*15:01g~DQB1*06:02g (frequency ranks 6, 9 and 13 in *CL-NI* and 4 and 10 in *CL-MP*) are included in the most frequent 6-locus haplotypes in all European reference populations in this study.

The association with alleles of other HLA genes in haplotypes also sheds light on the possible origin of the 4 LFD alleles that we identified as characteristic for our Mapuche sample. B*35:09 has also been documented in non-Chilean and non-Mapuche indigenous populations in South America, suggesting that the allele is virtually unique Native South American.

Apart from South American populations, B*39:09g occurs also in Asia but with other associations: In Asia, B*39:09-containing haplotypes show a conserved linkage of the allele to A*02:03 (64–66), in South American populations usually to A*02:01 or other HLA-A alleles. Also, linkage to DRB1 alleles is different in B*39:09-containing haplotypes from the two world regions. Common DRB1 allele associations like DRB1*15:01 or DRB1*04:05 in Asian haplotypes (65, 66) are not or very rarely seen in South American haplotypes. These differences suggest an independent origin of South American and Asian B*39:09 alleles.

For alleles DRB1*04:07g and DRB1*16:02g, both of which are present in virtually all Native populations from North, Central and South America, but also in Europe (DRB1*04:07g) and Asia (DRB1*16:02g), the examination of the DQB1 associations is distinctive. DRB1*04:07~DQB1*03:02 is a conserved Native American haplotype block while DRB1*04:07~DQB1*03:01 is found in Europeans (65, 67). Similarly, DRB1*16:02~DQB1*03:01 is found in Native Americans while DRB1*16:02~DQB1*05:02 is typically seen in Asians and Africans (61, 64). These different DRB1-DQB1 associations also indicate a possible convergent evolution of the alleles.

Overall, the cumulative frequency of haplotypes containing one or more of the four LFD alleles that we identified as characteristic for our Mapuche sample is 31.8% in *CL-MP* and still 20.9% in *CL-NI*. Given this high percentage even among those that self-identify as “Non-indigenous”, it is reasonable to assume that Chilean patients would benefit most from donors with similarly mixed European-Native South American ancestry and vice versa. Because of specific alleles that are rare outside South America, donors from the Hispanic/Latino groups in the United States with predominantly Mexican, Central American and Caribbean ancestry (6) may not be a match for Chilean or South American patients.

In the MP analysis, it is apparent that non-Chilean patients benefit more from a Chilean donor registry (approximated in this work by a registry of non-Indigenous Chilean donors, because they represent by far the majority of donors in the existing Chilean registry) than Chilean patients (non-Indigenous and Mapuche) benefit from registries of non-Chilean donors. This effect is particularly pronounced in the European reference populations, but also applies to our South American reference populations. This observation suggests a strong need for a Chilean donor registry to serve Chilean patients.

However, caution is warranted in interpreting our MP results, mainly for two reasons: First, the results are based on fairly small samples of size *n*=1,000, an approach we intentionally chose to avoid bias due to different sample sizes and to be able to include as many reference populations as possible in the analyses. We expect to considerably underestimate the real MP given the small samples, although the order of MP values between the different populations should be less affected. Second, we only considered matching probabilities for one donor and one patient population each. For a more realistic modeling of actual donor searches, one would need to construct a donor pool from numerous populations (42). We refrained from doing so because of the limitations posed by the small sample sizes anyway and the different main focus of this work.

In the MP context, it is also interesting to look at the actual stem cell collections for unrelated transplantation from Chilean donors and for Chilean patients (68). In 2021, there were 50 collections from Chilean donors, 33 for Chilean patients, and 17 for patients from 8 other countries. In addition, there were 35 donations from foreign donors for Chilean patients, of which the large majority were from Germany (*n*=26). Given that the Chilean donor registry represents less than 0.4% of the global donor pool, the fact that more foreign donors donated to Chilean patients than vice versa does not contradict our finding based on MP that non-Chilean patients generally benefit more from Chilean donors than vice versa. Also, the fact that the majority of non-domestic stem cell donations to Chilean patients came from Germany is probably due to the very large number of registered donors in Germany (>9M).

Apart from the above-mentioned constraints in the context of matching probabilities, our study is subject to further limitations that could influence the results. First, registered stem cell donors may not constitute an unbiased sample of the respective populations under consideration. This is, for example, due to the fact that their age distribution differs from that of the total population, since only persons aged 18-60 can be registered donors and the recruitment efforts are focused on young donors. Younger subjects in particular are likely to have higher genotype diversity due to a change in mobility and mating patterns over the generations. Furthermore, individuals of higher socioeconomic status may be more likely to be reached by invitations to register. In addition, it can be assumed that due to the peculiarities of the recruitment process, the regional distributions of registered donors and the total population do not match. For example, the nearly 50% of donors in our sample residing in the Santiago metropolitan region exceed the regional population share (~36%). It is known that the regional composition of the Chilean population varies (17, 69), so that, for example, a successful donor drive in a small town may create bias. Since the donor’s current address is not a good proxy of regional differences due to high mobility of the young target group, we refrained from stratification based on this parameter in our analyses. Second, the assignment of registered donors to individual populations is based on self-assessed ancestry during the recruitment process. This may not always result in consistent information, especially for donors of mixed ethnicity. Third, different levels of resolution limit the comparability of alleles between and with published data. In our study, we confined allele comparisons to a level of resolution that differentiates non-synonymous mutations of the antigen recognition site, neglecting possible variations outside this gene region. And finally, the fact that most reference population samples consist entirely of migrants and their descendants is a source of bias that is difficult to assess (70). For example, judging from their location in the MDS plot, the sample of Brazilian donors could be reflecting subpopulations with high European components and not necessarily the ethnic diversity represented in that country. Similarly, the sample of Hispanics from the United States is composed of individuals from very different genetic backgrounds. However, we have used the approach before and obtained conclusive results, so we consider it reasonable (71).

Similar to other DKMS registries, the proportion of female volunteer donors in DKMS Chile is high (70.8%). Gender imbalance is a common observation in stem cell donor registries worldwide (72). However, as the HLA alleles studied in our work are located on chromosome 6 of the human genome, we do not expect any bias for our results.

In summary, we analyzed HLA allele and haplotype frequency distributions of Chilean stem cell donors of non-Indigenous and Mapuche origin. With *n*=92,788 and *n*=1,993 individuals, respectively, these are the largest population samples of this type to date. We identified alleles with substantially higher frequencies in these two populations than in populations outside Chile. Our analyses of allele and haplotype frequencies, genetic distances and matching probabilities showed that both populations are quite similar as they carry an admixture of European and Native American HLA genes and haplotypes, thus reflecting the country’s history of admixture and immigration. Nevertheless, when comparing the two samples, we found 4 alleles with a Native American origin with high specificity for the Mapuche population, namely B*39:09g, B*35:09, DRB1*04:07g and DRB1*16:02g. Our results regarding matching probabilities, suggest a particular need for more stem cell donor recruitment in Chile, but need to be further confirmed due to the small samples and the simple model used.

## Data availability statement

The datasets presented in this article are not readily available because of ethical and privacy restrictions. Requests to access the datasets should be directed to the corresponding author/s.

## Ethics statement

Ethical review and approval was not required for the study on human participants in accordance with the local legislation and institutional requirements. Written informed consent for participation was not required for this study in accordance with the national legislation and the institutional requirements. DNA extraction from blood samples or buccal mucosal swabs was performed with the informed consent of the donors. The consent allowed the processing of anonymized donor data for research related to donor search or stem cell donation. The publication itself does not contain any identifiable personal data.

## Author contributions

US, AS and JS designed the study. AG and MG were responsible for stem cell donor recruitment and administration. VL was responsible for donor HLA typing. US organized the database and carried out analyses. UVS and AHS wrote the first draft of the manuscript with support from MF-V. All authors contributed to the article and approved the submitted version.

## References

[1] Passweg JR Baldomero H Chabannon C Basak GW de la Camara R Corbacioglu S. Hematopoietic cell transplantation and cellular therapy survey of the EBMT: monitoring of activities and trends over 30 years. Bone Marrow Transplant (2021) 56(7):1651–64. doi: 10.1038/s41409-021-01227-8 PMC8263343 33623153

[2] Dehn J Spellman S Hurley CK Shaw BE Barker JN Burns LJ. Selection of unrelated donors and cord blood units for hematopoietic cell transplantation: guidelines from NMDP/CIBMTR. Blood (2019) 134(12):924–34. doi: 10.1182/blood.2019001212 PMC6753623 31292117

[3] Lee SJ Klein J Haagenson M Baxter-Lowe LA Confer DL Eapen M. High-resolution donor-recipient HLA matching contributes to the success of unrelated donor marrow transplantation. Blood (2007) 110(13):4576–83. doi: 10.1182/blood-2007-06-097386 17785583

[4] Pidala J Lee SJ Ahn KW Spellman S Wang HL Aljurf M. Nonpermissive HLA-DPB1 mismatch increases mortality after myeloablative unrelated allogeneic hematopoietic cell transplantation. Blood (2014) 124(16):2596–606. doi: 10.1182/blood-2014-05-576041 PMC4199961 25161269

[5] Barker DJ Maccari G Georgiou X Cooper MA Flicek P Robinson J. The IPD-IMGT/HLA database. Nucleic Acids Res (2023) 51(D1):D1053–D60. doi: 10.1093/nar/gkac1011 PMC9825470 36350643

[6] Fernandez Vina MA Hollenbach JA Lyke KE Sztein MB Maiers M Klitz W. Tracking human migrations by the analysis of the distribution of HLA alleles, lineages and haplotypes in closed and open populations. Philos Trans R Soc Lond B Biol Sci (2012) 367(1590):820–9. doi: 10.1098/rstb.2011.0320 PMC3267126 22312049

[7] Meyer D Thomson G. How selection shapes variation of the human major histocompatibility complex: a review. Ann Hum Genet (2001) 65(Pt 1):1–26. doi: 10.1046/j.1469-1809.2001.6510001.x 11415519

[8] Meyer D Aguiar VRC Bitarello BD Brandt DYC Nunes K. A genomic perspective on HLA evolution. Immunogenetics (2018) 70(1):5–27. doi: 10.1007/s00251-017-1017-3 28687858 PMC5748415

[9] Single RM Meyer D Nunes K Francisco RS Hunemeier T Maiers M. Demographic history and selection at HLA loci in native americans. PloS One (2020) 15(11):e0241282. doi: 10.1371/journal.pone.0241282 33147239 PMC7641399

[10] WMDA. World marrow donor association . Available at: https://wmda.info/ (Accessed February 16, 2023).

[11] Jaimovich G Martinez Rolon J Baldomero H Rivas M Hanesman I Bouzas L. Latin America: The next region for haematopoietic transplant progress. Bone Marrow Transplant (2017) 52 (5) 671–7. doi: 10.1038/bmt.2016.361 28112744

[12] Arrieta-Bolanos E Oliveira DC Barquera R. Differential admixture, human leukocyte antigen diversity, and hematopoietic cell transplantation in Latin America: challenges and opportunities. Bone Marrow Transplant (2020) 55(3):496–504. doi: 10.1038/s41409-019-0737-4 31695172

[13] Jaimovich G Gale RP Hanesman I Rolon JM. The state of haematopoietic cells transplantation in Latin America. Lancet Haematol (2021) 8(1):e20–e1. doi: 10.1016/S2352-3026(20)30410-5 33357478

[14] Correa C Gonzalez-Ramella O Baldomero H Basquiera AL Baena R Arcuri L. Increasing access to hematopoietic cell transplantation in Latin America: results of the 2018 LABMT activity survey and trends since 2012. Bone Marrow Transplant (2022) 57(6):881–8. doi: 10.1038/s41409-022-01630-9 35347244

[15] Instituto nacional de estadísticas (INE) Chile, estimaciones y proyecciones de la población de Chile 1992-2050 (Total país) (2017). Available at: http://www.censo2017.cl/ (Accessed February 21, 2023).

[16] Bundeszentrale für politische bildung; länderprofile migration: migration in Chile . Available at: https://www.bpb.de/gesellschaft/migration/laenderprofile/suedamerika/328131/chile (Accessed Oktober 28, 2021).

[17] Eyheramendy S Martinez FI Manevy F Vial C Repetto GM. Genetic structure characterization of chileans reflects historical immigration patterns. Nat Commun (2015) 6:6472. doi: 10.1038/ncomms7472 25778948 PMC4382693

[18] Disi Pavlic R. Explaining outcomes of asymmetric conflicts revisited: the arauco war. Estudios Internacionales (2018) 50(189):97–119. doi: 10.5354/0719-3769.2018.49062

[19] Parvex R. Le chili et les mouvements migratoires. Hommes Migrations (2014) 1305):71–6. doi: 10.4000/hommesmigrations.2720

[20] Servicio nacional de migraciones gobierno de Chile, estimación de personas extranjeras (2021). Available at: https://serviciomigraciones.cl/estadisticasmigratorias/estimacionesdeextranjeros/ (Accessed February 16, 2023).

[21] Latinobarómetro - informe Chile (2020). Available at: https://www.latinobarometro.org (Accessed October 28, 2021).

[22] Arnaiz-Villena A Parga-Lozano C Moreno E Areces C Rey D Gomez-Prieto P. The origin of amerindians and the peopling of the americas according to HLA genes: admixture with Asian and pacific people. Curr Genomics (2010) 11(2):103–14. doi: 10.2174/138920210790886862 PMC2874220 20885818

[23] Arnaiz-Villena A Juarez I Lopez-Nares A Palacio-Gruber J Vaquero C Callado A. Frequencies and significance of HLA genes in amerindians from Chile canete mapuche. Hum Immunol (2019) 80(7):419–20. doi: 10.1016/j.humimm.2019.04.015 31101374

[24] Rey D Parga-Lozano C Moscoso J Areces C Enriquez-de-Salamanca M Fernandez-Honrado M. HLA genetic profile of mapuche (Araucanian) amerindians from Chile. Mol Biol Rep (2013) 40(7):4257–67. doi: 10.1007/s11033-013-2509-3 23666052

[25] Schäfer C Sauter J Riethmuller T Kashi ZM Schmidt AH Barriga FJ. HLA-A, -B, -DRB1 allele and haplotype frequencies of 920 cord blood units from central Chile. Hum Immunol (2016) 77(8):622–3. doi: 10.1016/j.humimm.2016.05.020 27233642

[26] Castro-Santos P Olloquequi J Diaz-Pena R. HLA-A, B, C and DRB1 alleles in a Chilean population from talca. HLA (2020) 95(3):200–3. doi: 10.1111/tan.13775 31846232

[27] Lange V Böhme I Hofmann J Lang K Sauter J Schöne B. Cost-efficient high-throughput HLA typing by MiSeq amplicon sequencing. BMC Genomics (2014) 15:63. doi: 10.1186/1471-2164-15-63 24460756 PMC3909933

[28] Schöfl G Lang K Quenzel P Böhme I Sauter J Hofmann JA. 2.7 million samples genotyped for HLA by next generation sequencing: lessons learned. BMC Genomics (2017) 18(1):161. doi: 10.1186/s12864-017-3575-z 28196473 PMC5309984

[29] Schäfer C Schmidt AH Sauter J. Hapl-o-Mat: open-source software for HLA haplotype frequency estimation from ambiguous and heterogeneous data. BMC Bioinf (2017) 18(1):284. doi: 10.1186/s12859-017-1692-y PMC5450239 28558647

[30] Solloch UV Schmidt AH Sauter J. Graphical user interface for the haplotype frequency estimation software hapl-o-Mat. Hum Immunol (2022) 83(2):117–2. doi: 10.1016/j.humimm.2021.11.002 34799151

[31] Excoffier L Slatkin M. Maximum-likelihood estimation of molecular haplotype frequencies in a diploid population. Mol Biol Evol (1995) 12(5):921–7. doi: 10.1093/oxfordjournals.molbev.a040269 7476138

[32] Schmidt AH Baier D Solloch UV Stahr A Cereb N Wassmuth R. Estimation of high-resolution HLA-A, -B, -C, -DRB1 allele and haplotype frequencies based on 8862 German stem cell donors and implications for strategic donor registry planning. Hum Immunol (2009) 70(11):895–902. doi: 10.1016/j.humimm.2009.08.006 19683023

[33] Lewontin RC. The interaction of selection and linkage. i. general considerations; heterotic models. Genetics (1964) 49:49–67. doi: 10.1093/genetics/49.1.49 17248194 PMC1210557

[34] Nothnagel M Fürst R Rohde K. Entropy as a measure for linkage disequilibrium over multilocus haplotype blocks. Hum Hered (2002) 54(4):186–98. doi: 10.1159/000070664 12771551

[35] Guo SW Thompson EA. Performing the exact test of hardy-Weinberg proportion for multiple alleles. Biometrics (1992) 48(2):361–72. doi: 10.2307/2532296 1637966

[36] Excoffier L Lischer HE. Arlequin suite ver 3.5: a new series of programs to perform population genetics analyses under Linux and windows. Mol Ecol Resour (2010) 10(3):564–7. doi: 10.1111/j.1755-0998.2010.02847.x 21565059

[37] Sullivan GM Feinn R. Using effect size-or why the p value is not enough. J Grad Med Educ (2012) 4(3):279–82. doi: 10.4300/JGME-D-12-00156.1 PMC3444174 23997866

[38] Klitz W Stephens JC Grote M Carrington M. Discordant patterns of linkage disequilibrium of the peptide-transporter loci within the HLA class II region. Am J Hum Genet (1995) 57(6):1436–44. PMC1801434 8533774

[39] Cavalli-Sforza LL Edwards AW. Phylogenetic analysis. models and estimation procedures. Am J Hum Genet (1967) 19(3 Pt 1):233–57. PMC1706274 6026583

[40] R Core Team. R: a language and environment for statistical computing (2022). Vienna, Austria: R Foundation for Statistical Computing. Available at: https://www.r-project.org/ (Accessed January 9, 2022).

[41] Müller CR Ehninger G Goldmann SF. Gene and haplotype frequencies for the loci HLA-A, HLA-B, and HLA-DR based on over 13,000 german blood donors. Hum Immunol (2003) 64(1):137–51. doi: 10.1016/s0198-8859(02)00706-1 12507825

[42] Schmidt AH Sauter J Pingel J Ehninger G. Toward an optimal global stem cell donor recruitment strategy. PloS One (2014) 9(1):e86605. doi: 10.1371/journal.pone.0086605 24497958 PMC3907384

[43] Turner EV Dilioglou S Arnold PY Palma J Rivera G. The HLA-A*68:23 allele in the Chilean population. Tissue Antigens (2014) 84(6):565–7. doi: 10.1111/tan.12462 25352173

[44] Hurley CK Kempenich J Wadsworth K Sauter J Hofmann JA Schefzyk D. Common, intermediate and well-documented HLA alleles in world populations: CIWD version 3.0.0. HLA (2020) 95(6):516–31. doi: 10.1111/tan.13811 PMC7317522 31970929

[45] Fallin D Schork NJ. Accuracy of haplotype frequency estimation for biallelic loci, *via* the expectation-maximization algorithm for unphased diploid genotype data. Am J Hum Genet (2000) 67(4):947–59. doi: 10.1086/303069 PMC1287896 10954684

[46] Hollenbach JA Thomson G Cao K Fernandez-Vina M Erlich HA Bugawan TL. HLA diversity, differentiation, and haplotype evolution in mesoamerican natives. Hum Immunol (2001) 62(4):378–90. doi: 10.1016/S0198-8859(01)00212-9 11295471

[47] Cano P Testi M Andreani M Khoriaty E Bou Monsef J Galluccio T. HLA population genetics: a Lebanese population. Tissue Antigens (2012) 80(4):341–55. doi: 10.1111/j.1399-0039.2012.01936.x 22994155

[48] Gonzalez-Galarza FF McCabe A Santos E Jones J Takeshita L Ortega-Rivera ND. Allele frequency net database (AFND) 2020 update: gold-standard data classification, open access genotype data and new query tools. Nucleic Acids Res (2020) 48(D1):D783–D8. doi: 10.1093/nar/gkz1029 PMC7145554 31722398

[49] Arnaiz-Villena A Palacio-Gruber J Juarez I Hernandez E Muniz E Bayona B. HLA in north Colombia chimila amerindians. Hum Immunol (2018) 79(4):189–90. doi: 10.1016/j.humimm.2018.02.004 29454071

[50] Paez-Gutierrez IA Hernandez-Mejia DG Vanegas D Camacho-Rodriguez B Perdomo-Arciniegas AM. HLA-A, -B, -C, -DRB1 and -DQB1 allele and haplotype frequencies of 1463 umbilical cord blood units typed in high resolution from bogota, Colombia. Hum Immunol (2019) 80(7):425–6. doi: 10.1016/j.humimm.2019.03.006 30862452

[51] Cerna M Falco M Friedman H Raimondi E Maccagno A Fernandez-Viña M. Differences in HLA class II alleles of isolated south American Indian populations from Brazil and Argentina. Hum Immunol (1993) 37(4):213–20. doi: 10.1016/0198-8859(93)90504-t 8300406

[52] Arrieta-Bolanos E Madrigal-Sanchez JJ Stein JE Orlich-Perez P Moreira-Espinoza MJ Paredes-Carias E. High-resolution HLA allele and haplotype frequencies in majority and minority populations of Costa Rica and Nicaragua: differential admixture proportions in neighboring countries. HLA (2018) 91(6):514–29. doi: 10.1111/tan.13280 29687625

[53] Shankarkumar U Pawar A Ghosh K Bajpai S Pazare A. Human leucocyte antigen class II DRB1 and DQB1 associations in human immunodeficiency virus-infected patients of Mumbai, India. Int J Immunogenet (2010) 37(3):199–204. doi: 10.1111/j.1744-313X.2010.00911.x 20345872

[54] Mack S Sanchez-Mazas A Tsai Y Erlich HA. 13th International histocompatibility workshop anthropology/human genetic diversity joint report - chapter 3: Anthropology/ human genetic diversity population reports, in: Hansen J, editor. Immunobiology of the Human MHC: Proceedings of the 13th International Histocompatibility Workshop and Conference. Seattle, USA: IHWG Press. (2006) pp. 580–652.

[55] Layrisse Z Guedez Y Domıínguez E Paz N Montagnani S Matos M. Extended HLA haplotypes in a carib amerindian population: the yucpa of the perija range. Hum Immunol (2001) 62(9):992–1000. doi: 10.1016/s0198-8859(01)00297-x 11543901

[56] Pimtanothai N Charoenwongse P Mutirangura A Hurley CK. Distribution of HLA-b alleles in nasopharyngeal carcinoma patients and normal controls in Thailand. Tissue Antigens (2002) 59(3):223–5. doi: 10.1034/j.1399-0039.2002.590308.x 12074714

[57] Chen S Ren X Liu Y Hu Q Hong W Xu A. Human leukocyte antigen class I polymorphism in miao, bouyei, and shui ethnic minorities of guizhou, China. Hum Immunol (2007) 68(11):928–33. doi: 10.1016/j.humimm.2007.09.006 18082574

[58] Trachtenberg EA Keyeux G Bernal JE Rhodas MC Erlich HA. Results of expedicion humana. i. analysis of HLA class II (DRB1-DQA1-DPB1) alleles and DR-DQ haplotypes in nine Amerindian populations from Colombia. Tissue Antigens (1996) 48(3):174–81. doi: 10.1111/j.1399-0039.1996.tb02625.x 8896175

[59] Leffell MS Fallin MD Hildebrand WH Cavett JW Iglehart BA Zachary AA. HLA alleles and haplotypes among the Lakota Sioux: report of the ASHI minority workshops, part III. Hum Immunol (2004) 65(1):78–89. doi: 10.1016/j.humimm.2003.10.001 14700599

[60] Gragert L Madbouly A Freeman J Maiers M. Six-locus high resolution HLA haplotype frequencies derived from mixed-resolution DNA typing for the entire US donor registry. Hum Immunol (2013) 74(10):1313–20. doi: 10.1016/j.humimm.2013.06.025 23806270

[61] Mack SJ Bugawan TL Moonsamy PV Erlich JA Trachtenberg EA Paik YK. Evolution of Pacific/Asian populations inferred from HLA class II allele frequency distributions. Tissue Antigens (2000) 55(5):383–400. doi: 10.1034/j.1399-0039.2000.550501.x 10885559

[62] Jinam TA Saitou N Edo J Mahmood A Phipps ME. Molecular analysis of HLA class I and class II genes in four indigenous Malaysian populations. Tissue Antigens (2010) 75(2):151–8. doi: 10.1111/j.1399-0039.2009.01417.x 20003135

[63] Chandanayingyong D Stephens HAF Klaythong R Sirikong M Udee S Longta P. HLA-a, -b, -DRB1, -DQA1, and -DQB1 polymorphism in thais. Hum Immunol (1997) 53(2):174–82. doi: 10.1016/s0198-8859(96)00284-4 9129976

[64] Maiers M Gragert L Klitz W. High-resolution HLA alleles and haplotypes in the united states population. Hum Immunol (2007) 68(9):779–88. doi: 10.1016/j.humimm.2007.04.005 17869653

[65] Pingel J Solloch UV Hofmann JA Lange V Ehninger G Schmidt AH. High-resolution HLA haplotype frequencies of stem cell donors in Germany with foreign parentage: how can they be used to improve unrelated donor searches? Hum Immunol (2013) 74(3):330–40. doi: 10.1016/j.humimm.2012.10.029 23200758

[66] Kwok J Tang WH Chu WK Chan YS Liu Z Yang W. High resolution allele genotyping and haplotype frequencies for NGS based HLA 11 loci of 5266 Hong Kong Chinese bone marrow donors. Hum Immunol (2020) 81(10-11):577–9. doi: 10.1016/j.humimm.2020.08.005 32893027

[67] Seitz S Lange V Norman PJ Sauter J Schmidt AH. Estimating HLA haplotype frequencies from homozygous individuals - a technical report. Int J Immunogenet (2021) 48(6):490–5. doi: 10.1111/iji.12553 PMC9131737 34570965

[68] WMDA global trends report (2021). Available at: https://wmda.info/about-us/media-centre/ (Accessed June 27, 2022).

[69] Parolin ML Toscanini UF Velazquez IF Llull C Berardi GL Holley A. Genetic admixture patterns in Argentinian Patagonia. PloS One (2019) 14(6):e0214830. doi: 10.1371/journal.pone.0214830 31206551 PMC6576754

[70] Solloch UV Lang K Lange V Böhme I Schmidt AH Sauter J. Frequencies of gene variant CCR5-Δ32 in 87 countries based on next-generation sequencing of 1.3 million individuals sampled from 3 national DKMS donor centers. Hum Immunol (2017) 78(11-12):710–7. doi: 10.1016/j.humimm.2017.10.001 28987960

[71] Sauter J Putke K Schefzyk D Pruschke J Solloch UV Bernas SN. HLA-E typing of more than 2.5 million potential hematopoietic stem cell donors: methods and population-specific allele frequencies. Hum Immunol (2021) 82(7):541–7. doi: 10.1016/j.humimm.2020.12.008 33386168

[72] Fingrut W Rikhraj K Allan D. Targeted recruitment of male donors for allogeneic haematopoietic cell transplantation: a review of the evidence. Vox Sang (2018) 113(4):307–16. doi: 10.1111/vox.12632 29359335

